# Interleukin-37 Ameliorates Articular Cartilage Damage in Two Murine Models of Osteoarthritis

**DOI:** 10.1177/19476035251372304

**Published:** 2025-09-19

**Authors:** Ellen W. van Geffen, Henk M. van Beuningen, Joyce Aarts, Elly L. Vitters, Wim H.C. Rijnen, Arjen B. Blom, Fons A.J. van de Loo, Esmeralda N. Blaney Davidson, Marije I. Koenders, Arjan P.M. van Caam, Peter M. van der Kraan

**Affiliations:** 1 Department of Experimental Rheumatology, Radboud University Medical Center, Nijmegen, The Netherlands; 2 Department of Orthopaedics, Radboud University Medical Center, Nijmegen, The Netherlands

**Keywords:** osteoarthritis, interleukin-37, cartilage damage, mouse, DMM model, CiOA model

## Abstract

**Objective:**

In this study, we investigated whether interleukin (IL)-37 ameliorates experimental osteoarthritis (OA).

**Methods:**

The effects of IL-37 were investigated in collagenase-induced OA (CiOA) and destabilization of the medial meniscus (DMM). Human IL-37-adenovirus (ad-IL-37) was injected into the affected knee joint 4 and 18 days after the induction of OA. Luciferase-adenovirus was injected as control. Mice were sacrificed for histology at early and late stage of OA development. Interleukin-37 protein expression was confirmed by immunohistochemistry. Cartilage damage, osteophyte size and joint capsule thickness were measured. The effectiveness of ad-IL-37 was confirmed in vitro in human OA fibroblasts using real-time qualitative polymerase chain reaction (RT-qPCR) and Western blotting.

**Results:**

Interleukin-37 protein expression was visible for at least 7 days after injection. At day 28, 10 days after the second injection, no clear synovial IL-37 staining was found any more, in both models. At day 28 of CiOA, ad-IL-37 significantly reduced articular cartilage damage and this was still reduced, although not significantly, at the late time point (day 42). In the DMM model, significant beneficial effect of IL-37 on cartilage damage was found at the late time point. In both OA models ad-IL-37 injections reduced the size of osteophytes.

**Conclusion:**

Interleukin-37 ameliorated OA-induced articular cartilage damage and osteophyte formation in both models.

## Introduction

Osteoarthritis (OA) is the most common joint disorder, primarily characterized by cartilage damage and osteophyte formation. Cartilage destruction varies from surface discontinuity, cartilage fibrillations, fissures, and erosion to even total loss.^1^ Concomitant with this injury, excess tissue production can be observed in the joint, resulting in osteophyte formation.^2^

Furthermore, in more than 50% of OA patients, synovitis is observed.^3-8^ In synovial fluid and serum of OA patients, presence of inflammatory mediators can be measured,^8-10^ and their levels positively correlate with cartilage damage in knee OA.^3,11^ Inflammatory factors can stimulate chondrocytes to produce cartilage degrading enzymes such as matrix metalloproteinases (MMPs), leading to cartilage damage.^12,13^ Damaged cartilage fragments (DAMPs) can in turn activate synoviocytes to produce inflammatory factors, resulting in a positive feedback loop between inflammation and cartilage damage.^7^ Interfering with this vicious cycle is therefore a potential strategy for OA treatment.

Potentially, application of interleukin-37 (IL-37) interferes with this vicious cycle. Interleukin-37 is a member of the IL-1 family with, in contrast to its famous pro-inflammatory family member IL-1β, potent anti-inflammatory and immunosuppressive properties.^14^ Previously, we have shown that IL-37 lowers cytokine and MMP production in chondrocytes,^15^ and protects articular cartilage against proteoglycan loss in vitro and ex vivo.^16^ Both intracellular and extracellular IL-37 can have these effects. Inside the cell, IL-37 can be produced in response to inflammatory stimuli and translocate to the nucleus, probably via binding to phosphorylated SMAD3 (pSMAD3), where it can inhibit the transcription of genes coding for inflammatory cytokines.^17^ In the extracellular space, IL-37 can bind to the IL-18 receptor α-chain (IL18Rα). This binding recruits the orphan IL-1 family receptor IL-1 receptor 8 (IL-1R8).^18^ In chondrocytes, this IL-37-IL-1R8-IL-18Rα complex inhibits p38, ERK, JNK, and NF-κB activation.^19^ In macrophages, binding of IL-37 inhibits activation of the NLRP3 inflammasome.^20^ So far, a homologous gene for IL-37 has not been identified in the mouse. However, growing evidence confirms the anti-inflammatory effects of exogenous, recombinant human IL-37 on wild-type mice. For example, administration of IL-37 protects mice against experimental colitis, liver inflammation and experimental arthritis *in vivo*.^21-23^ Protection by IL-37 against experimental arthritis (reduced cartilage damage and subchondral bone loss) was also found in rat temporomandibular joint.^19^ In this study, we investigated whether IL-37 also has a protective role in experimental OA, possibly by modulating the low-grade joint inflammation present in the models.

## Material and Methods

### Part A: In Vitro Check for Expression and Functionality of IL-37 Transduced via Adenoviral Constructs in Human OA Synovial Fibroblasts

#### OA synovial fibroblast culture

Synovial tissue was obtained from surgical waste tissue of 10 OA patients receiving knee joint replacement surgery. The tissue was digested using Liberase (50 µg/ml) (Roche, Basel, Switzerland) for 1 h at 37°C on a roller bench and subsequently filtered through a 70 µm cell strainer (Corning, NY, USA). Cells were spun down, washed and then cultured in Dulbecco’s Modified Eagle Medium (DMEM; Gibco) supplemented with 10% fetal calf serum (FCS), 100 mg/l pyruvate and 100 U/ml penicillin and 100 mg/ml streptomycin, in standard culture conditions (37°C, 5% CO_2_, 95% humidity). After 3 passages the cells were stored in liquid nitrogen for later use.

#### Fibroblast in vitro experiment

For gene expression studies, 1 × 10^5^ fibroblasts per well were seeded in 24-well culture plates and for protein expression studies, 1 × 10^6^ fibroblasts per well were seeded in 6-wells culture plate (Cell star, Greiner Bio-one). At 80% confluence, fibroblasts were transduced with an adenoviral vector (serotype 5 adenoviruses [Ad5] vectors), overexpressing human IL-37b (Ad-IL-37) or luciferase (Ad-Luc; control virus) for 3 h at 37°C in a multiplicity of infection (MOI) of 25 in serum-free medium.^15^ This MOI induces high IL-37 gene expression and protein production and secretion **(Suppl. Fig. S2)**. Subsequently, fibroblasts were serum-starved overnight and stimulated with IL1β (0-10 ng/ml) or human OA synovium-conditioned medium (OAS-CM) (0%-10%) for 6 h. Osteoarthritis synovium-conditioned medium is produced by culturing OA synovium pieces for 24 h in DMEM containing 0.1% bovine serum albumin (BSA). Hereafter, supernatant is collected, centrifugated to remove debris, aliquoted and stored at −20°C until further use.^24^

#### mRNA isolation and quantitative real-time PCR

Fibroblasts were dissolved in 500 µl TRI-reagent (Sigma-Aldrich) and RNA was isolated as previously described.^15^ RNA was reverse transcribed to cDNA and real-time qualitative polymerase chain reaction (RT-qPCR) was performed by using SYBR green master mix in the StepOnePlus real-time PCR system (Applied Biosystems) with 0.25 mM validated primers (Biolegio) (**Table 1**).^15^ Melting curves and water controls were included to verify product specificity. As reference genes *GAPDH* and *RPS27A* were used.

**Table 1.** Human Validated Primers Used for RT-qPCR.

Gene Symbol	Forward 5’→ 3’	Reverse 5’→ 3’
***ADAMTS5***	GCTCACGAAATCGGACATTTACTT	ACCAAGGTCTCTTCACAGAATTTG
***GAPDH***	ATCTTCTTTTGCGTCGCCAG	TTCCCCATGGTGTCTSGAGC
***IL1B***	AATCTGTACCTGTCCTGCGTGTT	TGGGTAATTTTTGGGATCTACACTCT
***IL37***	CCAAGCCTCCCCACCATGAA	TCCAGGACCAGTACTTTGTGATC
***IL6***	AGCCCACCGGGAACGA	GGACCGAAGGCGCTTGT
***IL8***	AGAAGTTTTTGAAGAGGGCTGAGA	AGTTTCACTGGCATCTTCACTGATT
***MMP1***	ACTGCCAAATGGGCTTGAAG	TTCCCTTTGAAAAACCGGACTT
***MMP3***	SGAGGCATCCACACCCTAGGTT	TCAGAAATGGCTGCATCGATT
***MMP13***	ATTAAGSGAGCATGGCGACTTCT	CCCAGSGAGGAAAAGCATSGAG
***RPS27A***	GTTAAGCTGGCTGTCCTGAAA	CATCAGAAGGGCACTCTCG

#### Protein isolation and western blot

Cell lysates were obtained as previously described^15^ and protein concentrations were equalized. Proteins were separated on a 10% reducing bisacrylamide SDS-PAGE gel and transferred onto a 0.1 µm pore nitrocellulose membrane using wet transfer (Towbin buffer, 2 h, 275 mA at 4°C). Subsequently, membranes were blocked and incubated over night at 4°C with primary antibodies against IL-37 (AF1975, 1:1500, R&D), followed by 1 h incubation with HRP labeled rabbit anti-goat antibody (1:1500, DAKO Belgium). Enhanced chemiluminescence using ECL prime kit (GE Healthcare, UK) was used to visualize proteins with the ImageQuant LAS4000 (Leica, Germany). GAPDH (1:10000, clone 1G5, Sigma-Aldrich) was used as loading control.

### Part B: Use of IL-37 Adenoviral Constructs In Vivo in Two Models of OA

#### Animals

The Dutch Central Commission on Animal Experiments approved all procedures involving animals (CCD #2017-0012). CiOA (protocol 2017-0012-002) was induced in 10 weeks old female C57BL/6NRj (Janvier) mice, and DMM (protocol 2017-0012-005) in 10 weeks old male C57BL/6NRj (Janvier) mice, because this model does not run efficiently in female mice.^25^ Mice were housed in filter-top cages with woodchip bedding and cage enrichment (igloo and shredded paper) under standard pathogen-free conditions. Water and standard diet were provided ad libitum. All mice were individually identified by an ear punch and treatment groups were allocated using a randomization tool (Random.org). Female mice were divided along cages in groups of six animals per cage, with each cage containing three mice from the IL-37 treatment group and three mice from the Luc-control group. To minimalize fighting, the males were housed in smaller groups (four animals/cage, two from each experimental group). For the same reason, the males received extra shelter (2 igloos) and double amount of shredded paper.

#### Animal experiments

Ad-IL-37 functionality in vivo was confirmed (protocol 2017-12-001) by injecting it intra-articularly in the right knee joint of naïve female mice (0.5x10^7^ plaque-forming units [pfu] in 6 µl 0.9% NaCl) followed by analysis of IL-37 protein expression on histological sections of joints at day 1, 3, 7, 14, 21 and 28 after injection. The control groups received Ad-Luc. Besides Il-37 expression, histology was also checked for possible effects of adenoviral constructs on joint structures. Per experimental group 4 mice were used, leading to a total number of 48 mice in this experiment.

Next, the effect of IL-37 on OA pathology was investigated in both OA models. We chose to use different timing of observation between the models since OA development in the two models has different kinetics. Osteoarthritis was induced in the right knee joint of the mice. No person involved in the execution of the animal experiments was aware of the treatments the experimental groups received. Substances A and B were injected. We started with the collagenase-induced OA model,^26^ which combines joint instability with inflammation in its early stages. Briefly, OA was induced by a single intra-articular injection of 3 units of bacterial collagenase type VII (Sigma-Aldrich) in 6 µl 0.9% NaCl. In a separate experiment the DMM was induced by cutting the ligament that attaches the frontal part of the medial meniscus to the tibia, the medial meniscotibial ligament, via a small incision of the joint capsule.^27^ Ketamine/xylazine anesthetics was used during the DMM operations, but no post-operative analgesics were used, because these could influence inflammation, and thereby possibly also OA development. All intra-articular injections were performed under 2.5% isoflurane inhalation anesthetics. Four days after induction of each OA model, Ad-IL-37 or Ad-Luc (0.5x10^7^ pfu) was intra-articularly injected into the affected knee joint. Two weeks later, injection with Ad-IL-37 or Ad-Luc was repeated. Mice were sacrificed on day 7, 28 and 42 after collagenase-injection or on days 28 and 56 after DMM induction. Group sizes were calculated based on earlier experiments in the models. We used the Russ Lenth power calculator app, with 2-sample *t*-test and correction for multiple testing. For the CiOA this resulted in a minimum group size of 12 mice (standard deviation 6.8, predicted effect size 9.1) and for the DMM in a minimum group size of 14 mice (standard deviation 5.8, predicted effect size 7,3). In the CiOA 60 female mice were used and in the DMM model 56 males, of which 5 were lost due to fighting. For an overview of the experimental design see **Suppl. Figure S1**.

#### Histology

Dissected knee joints of mice were fixed in 4% phosphate buffered formalin for 7 days, decalcified in 10% formic acid for 1 week, and subsequently dehydrated with an automated tissue processing apparatus and embedded in paraffin. Frontal sections of 7 µm were made. Deparaffinized sections were stained with hematoxylin/eosin (HE) or safranin-O/Fast Green (0.1% w/v in water). Analyses were performed in 5 sections per joint, with an interval of 140 µm in between. The scoring of pathology in histological sections was done in a blinded fashion (under code). Presence of growth plate on both lateral and medial side was used as starting landmark. Joint capsule thickening was measured in a blinded way, using Leica application suite V4.3 (Leica Switzerland) on three different locations per HE-stained section at both the lateral and the medial joint side, to account for possible variability, and an average per joint was calculated. Cartilage damage and osteophyte size were scored in a blinded way in safranin-O/Fast green-stained sections. Cartilage damage in the tibiofemoral joints was scored for the medial and lateral side of both the tibia and the femur using a more detailed version of the OARSI scoring system^28^ that is also based on the work of Pritzker *et al*.^1^ Our version has been described before.^29^ Briefly, this OA score represents the assessment of the OA grade (the severity of OA pathology observed; 0-6, where 0 represents no damage and 6 represents bone loss/remodeling/serious deformation of the subchondral bone) multiplied (as recommended by Pritzker *et al*.^1^) by the OA stage (showing the extent of OA as percentage of total surface that shows any degree of degeneration; 0-5, where 0 represents no OA activity observed and 5 represents >75% of the total surface area). This score (0-30) was measured in five histological sections per knee joint after which the average score per cartilage region per mouse was calculated. Graphs show the average individual scores per cartilage site (lateral femur, medial femur, lateral tibia, medial tibia). Osteophyte surface area was measured with Leica application suite V4.3 (Leica Switzerland) at medial tibia, medial femur, attachment of collateral ligament and medial patella groove. Of note, in the CiOA some mice with joint dislocations (day 28: 3 dislocations in Luc group [n = 12], 2 dislocations in IL-37 group (n = 12); day 42: 1 dislocation in Luc group (n = 12), 4 dislocations in IL-37 group (n = 12) were excluded from analysis.

Knee sections were immunohistochemically stained for IL-37 (1:400, ab116282, Abcam). Sections were deparaffinized and incubated with 1% hydrogen peroxide in methanol for 30 min to block endogenous peroxidases. Thereafter, sections were incubated with citrate buffer (0.1 M sodium citrate and 0.1 M citric acid) for 2 hours and blocked with 5% goat serum for 1 h at RT. Primary antibody against IL-37 was incubated overnight at 4°C. After washing with phosphate-buffered saline (PBS), biotinylated goat anti-rabbit (Dako) was used as secondary antibody, followed by incubation with horseradish peroxidase-conjugated streptavidin (Elite kit; Vector Laboratories) and diaminobenzidine (DAB). Sections were counterstained with hematoxylin, dehydrated and mounted.

#### Statistical analysis

Statistics were performed with Graphpad Prism version 10.1.2 (Graphpad Software, Inc., San Diego, CA, USA) with a Mann-Whitney *U*-test for read-out with ordered categorical variables, or unpaired Student-test for read-out with continuous variables. The in vivo experiment was designed for cartilage damage as single parameter. Cartilage damage in the knee joint was measured at four different sites (lateral femur, medial femur, lateral tibia, medial tibia). We decided to perform statistical analysis of articular cartilage damage only for the joint site with the highest damage in the ad-Luc group (medial femur in CiOA and medial tibia in DMM). The effects we describe for cartilage damage at the other three sites and for osteophyte size, another hallmark of OA, are not statistically analyzed and are at least observed trends. Therefore we did not have to correct for multiple comparisons.

Histology of one mouse in the d56 DMM adIL-37 group was not included in the analysis because this showed extreme pathology and was a significant outlier both for cartilage damage and osteophyte size (Grubbs test, *P* < .01 for both parameters). For in vitro studies of the effects of IL-37 in OA fibroblast of multiple donors, 1-way repeated measures analysis of variance (ANOVA) with Bonferroni multiple comparison post hoc test was used. Differences were considered significant when *P* value was <.05.

## Results

In his section of the manuscript, we want to have the focus on the in vivo OA models. The in vitro data are additive, to show functionality of the adenoviral constructs, also in human synovial fibroblast. For this reason, we will start with the results of the animal experiments.

### Human IL-37 Protein Does not Induce Observable Pathology in Murine Knee Joints

**Figure 1.**
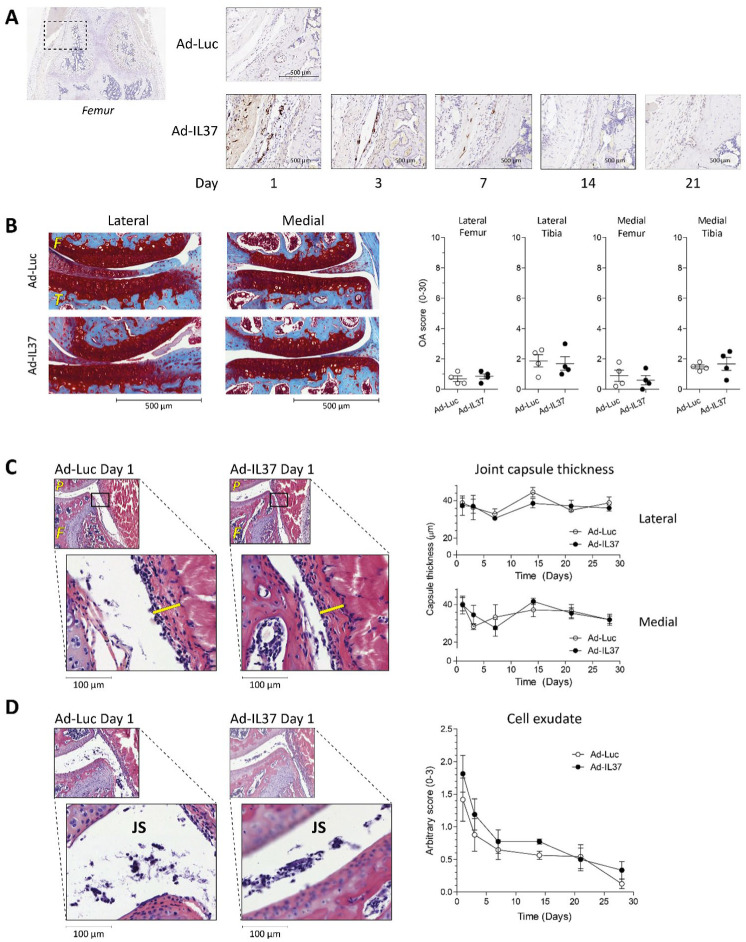
Human IL-37 protein is expressed and does not induce structural pathology in murine knee joints. **(A)** IL-37 protein expression in the synovial lining of knee joints of C57BL/6NRj mice after single intra-articular injection with IL-37-adenovirus as determined by immunohistochemistry. Luc-adenovirus was used as control. **(B)** Cartilage damage at day 28 was assessed on safranin-O/fast green stained histological sections of right knee joints using the modified OARSI score. **(C)** Joint capsule thickness (yellow bars) was measured on hematoxylin/ eosin (HE) stained histological sections. **(D)** Cell exudate was scored on an arbitrary scale from 0 to 3, where 0 = none and 3 = severe, on hematoxylin/eosin (HE)-stained histological sections. Representative pictures of histology are shown. Data are presented as mean ± S.E.M. with each symbol representing one mouse in Figure 1B, n = 4 mice per experimental group. F = femur; T = tibia; P = patella; JS = joint space.

Before investigating the effect of IL-37 on pathology in OA models, we first investigated if we could overexpress IL-37 in a murine knee joint using an adenovirus. After a single intra-articular injection, IL-37 protein was clearly expressed in synoviocytes up to day 7 as detected by immunohistochemistry (**Fig. 1A**). After 14 days, IL-37 staining was much less. Therefore, in the OA models (slowly developing pathology), we decided to give a second intra-articular injection with the IL-37-adenovirus 2 weeks after the first injection to maintain IL-37 levels during 1 month. Importantly, no staining for IL-37 was present in the Luc-injected knee joints.

Subsequently, we checked whether human IL-37 protein is well tolerated and does not cause visible pathology in murine knee joints. For this reason, we scored cartilage damage and inflammation by joint capsule thickness and cell exudate in adIL-37 and adLuc-control injected knee joints. Up to 28 days after injection, no differences in pathology between IL-37 and Luc-control were observed for all time points based on these parameters (**Fig. 1B-D**).

### IL-37 is Expressed and Reduces Joint Capsule Thickness on Day 7 of the CiOA-Model

**Figure 2.**
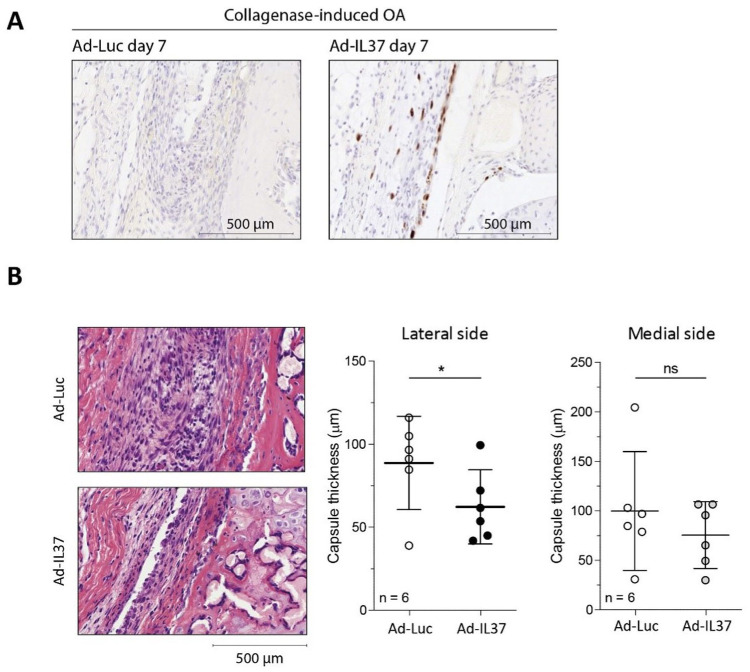
Treatment of collagenase-induced OA with-IL-37 significantly reduces joint capsule thickness at day 7. CiOA was induced in C57BL/6NRj mice and treatment with IL-37 adenovirus was started four days later. **(A**) IL-37 protein expression in the synovial lining of C57BL/6NRj mice knee joints intra-articularly injected with IL-37- or Luc-adenovirus, on day 7 after induction of OA, as determined by immunohistochemistry. (**B**) Joint capsule thickening on day 7 was measured both at lateral and medial joint side of knee joints intra-articularly injected with IL-37- or Luc-adenovirus. In both **A** and **B** the pictures of the tissues show only the lateral side of the joint. Data are presented as individual data points with mean ± 95% confidence intervals, n = 6 mice per experimental group; *= *P* < .05 as determined by unpaired Student’s t-test.

Next, we investigated whether under inflammatory conditions, early in the CiOA model, adIL-37 injection leads to IL-37 protein expression and functionality (reduction of joint inflammation). First, we confirmed the presence of IL-37 by immunohistochemistry. On day 7 after OA induction (3 days after intra-articular injection of adenoviral constructs) strong IL-37 protein staining was found in the synovial lining, thereby confirming its presence (**Fig. 2A**). Because this model is characterized by presence of joint inflammation, we measured joint capsule thickening on day 7 (**Fig. 2B**). Notably, IL-37 reduced joint capsule thickness with 30% on the lateral side. However, only a nonsignificant reduction was observed on the medial side.

### IL-37 Reduces Articular Cartilage Degradation and Osteophyte Formation on Day 28 After Induction of CiOA, but it does not Protect Articular Cartilage in Early DMM

**Figure 3.**
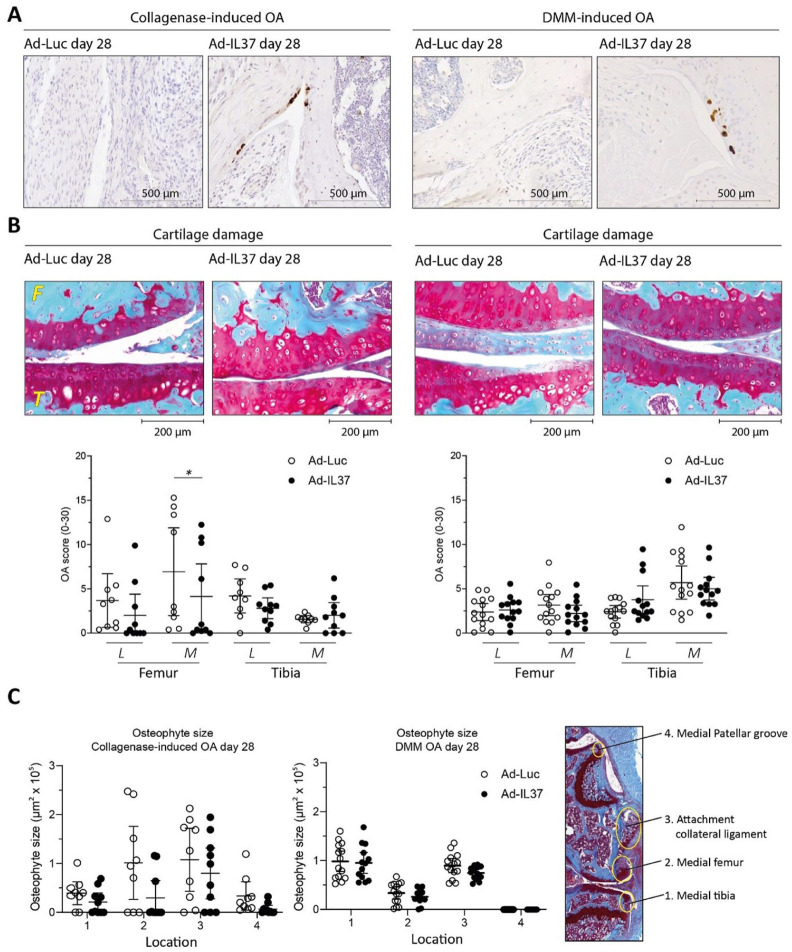
IL-37 reduces cartilage degradation on day 28 in the CiOA-model. Moreover, it reduces osteophyte formation in both CiOA and DMM at this time-point. OA was induced in C57BL/6NRj mice and IL-37 adenovirus was injected on day 4 and 18 after induction in both OA models. (**A**) IL-37 protein expression in C57BL/6NRj mice knee joints intra-articularly injected with IL-37- or Luc-adenovirus on day 28 after induction of OA, as determined by immunohistochemistry. At this time-point some positive cells were still present at specific sites in the central part of the femoro-tibial joint, in both models. However, no IL-37 protein was found in the synovium lining the joint capsule any more, in both OA models (not shown). (**B**) Cartilage damage was assessed on safranin-O/fast green-stained histological sections (five sections per mouse) using the modified OARSI score. Representative pictures of histological sections of the medial part of the joint are added. Again Collagenase-induced OA is placed at the left and DMM-induced OA at the right. (**C**) Osteophyte formation at 4 sites on the medial side of the knee joint was measured with image analysis software on Safranin-o/fast green-stained histological sections. Data are presented as individual data points with mean ± 95% confidence intervals, n = 9-10 mice per experimental group in CiOA (mice with dislocations were excluded from analysis) and n = 12 mice per experimental group in DMM. *= *P* < .05 as determined by Mann-Whitney *U*-test or unpaired Student’s *t*-test (statistical analysis was not performed for osteophyte size and for cartilage damage it was performed only for the site with the highest damage in the control group, this is explained in the section Statistical analysis). L = lateral; M = medial; F = femur; T = tibia.

Knowing that IL-37 injection does no harm to the naïve joint, we next wanted to investigate if IL-37 can indeed ameliorate OA pathology *in vivo* in experimental OA. The first time-point we chose for scoring of OA pathology was day 28. First, IL-37 protein expression was again confirmed by immunohistochemistry. At day 28, 10 days after the second injection of adenoviral constructs, synovial IL-37 protein was hardly visible in both models. Presence of stained cells was more confined to the femoro-tibial joint area (**Fig. 3A**). This could be expected from **Figure 1**, showing that synovial IL-37 expression in a naïve knee joint was already lost after 14 days. In the CiOA, the mean cartilage degradation was reduced by IL-37 treatment at the lateral and medial femur by 46 and 41%, respectively (**Fig. 3B**). As determined beforehand, see statistical analysis section, statistical significance of this reduction was only tested for the cartilage of the medial femur (*P* = .0467*)*. Our group size was determined for measurement of only one parameter, but the reader can also get an impression of effects measured in other cartilage sites and on osteophyte development. However, those results are purely observational. In DMM no effect on articular cartilage degradation was found at the early time-point with still little damage. In addition, osteophyte size was measured in the medial part of the knee joint, where osteophytes were most pronounced. (**Fig. 3C**). In the CiOA model, IL-37-treated knee joints showed reduced osteophyte size at the medial femur and medial patellar groove by 71% and 80%, respectively. In the DMM model, IL-37 treatment reduced osteophyte size only at the attachment site of the collateral ligament (17% reduction). Joint capsule thickness was not modulated by IL-37 at this time point in both OA models (data not shown). These results show that IL-37 can reduce the main hallmarks of OA pathology, that is, cartilage degradation and osteophyte formation, on day 28 after onset of experimental OA.

### Decreased IL-37-Effects on OA Pathology After 6 Weeks (CiOA) or 8 Weeks (DMM)

**Figure 4.**
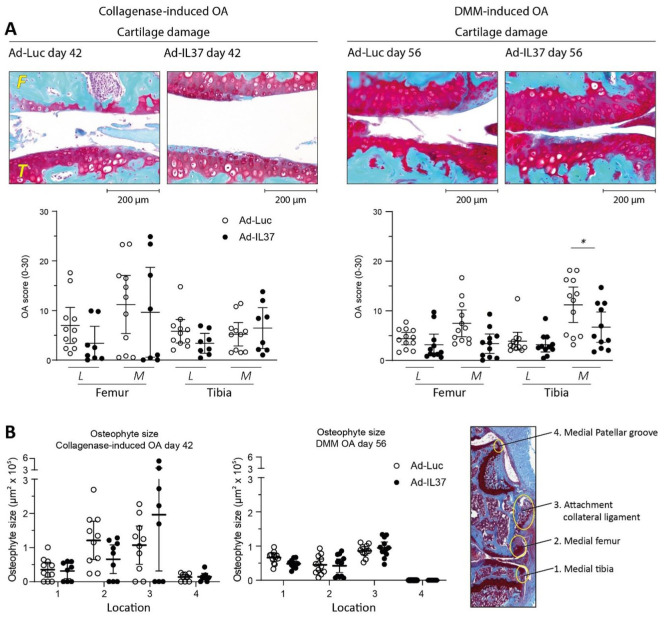
Early IL-37 treatment reduces cartilage damage in DMM at 8 weeks an in CiOA at 6 weeks. Moreover, IL-37 still clearly reduces osteophyte size in both models at these late time-points. C57BL/6NRj mice were injected with IL-37 adenovirus on days 4 and 18 after induction of OA in both models. (**A**) Cartilage damage was assessed on safranin-O/Fast green-stained histological sections using the modified OARSI score. Representative pictures of histological sections of the medial part of the joint are added. (**B**) Osteophyte formation at 4 sites on the medial side of the knee joint was measured with image analysis software on safranin-o/Fast green-stained histological sections. Data are presented as individual data points with mean ± 95% confidence intervals, n = 8-11 mice per experimental group in CiOA (mice with dislocations were excluded from analysis) and n = 13-14 mice per experimental group in DMM. *= *P* < .05 as determined by Mann Whitney *U*-test or unpaired Student’s *t*-test (statistical analysis was not performed for osteophyte size and for cartilage damage it was performed only for the site with the highest damage in the control group, this is explained in the section Statistical analysis). L = lateral; M = medial; F = femur; T = tibia.

Next, we investigated if the protective effects of IL-37 on OA pathology are present at a later time-point. In the CiOA-model, this was performed on day 42 after induction of the model. At this time point, 24 days after the second intra-articular injection, we no longer observed synovial IL-37 expression (data not shown). At the day 42 time-point, osteophyte size at the medial femur was still reduced by 46% by IL-37 (**Fig. 4B**). However, most likely due to the loss of IL-37 protein expression, cartilage damage at the medial femur was no longer significantly reduced by IL-37 treatment. In fact, at day 42 the mean cartilage damage at the medial femur almost reached the level of the control group. Despite the absence of statistically significant effects, we still found reduction of the mean cartilage damage at the lateral femur and lateral tibia of 52% and 43%, respectively (**Fig. 4A**)

In the DMM model the later time-point was chosen at day 56, because of the slower progression of this model. In the DMM model significant protection of articular cartilage was observed at the late time-point (**Fig. 4A**). Interleukin-37 reduced the mean cartilage damage on the medial femur and the medial tibia (55% and 40% reduction, respectively). As determined beforehand, in this model statistical significance of this reduction was tested only for the cartilage of the medial tibia (*P* = .0229). This is explained in the Statistical analysis section. If the cartilage damage in the, most affected, medial part of the joint would be taken together (medial femur + medial tibia), the *P* value would even reach .0085. Moreover, osteophyte size at the medial tibia was reduced by 27% at day 56 (**Fig. 4B**). Like day 28, also at these later time-points in both models no differences in joint capsule thickness were observed between IL-37- and Luc-treated knee joints (data not shown). Our results show difference in the modulation of cartilage damage by IL-37 in experimental OA models. In the DMM the protective effect of IL-37 is seen at day 56, long after the loss of IL-37 protein expression, while in the CiOA the protective effect is lost at day 42. However, clear reduction of osteophyte size is still found at the late time-point in both models. Cross-sectional analyses show that IL-37 clearly reduced osteophyte size at the medial femur and medial patellar groove (CiOA) and collateral ligament (DMM) at 4 weeks and reduced osteophyte size at the medial femur (CiOA) and medial tibia (DMM) at later time points. Worth mentioning here: cartilage damage appears to increase between early and late time-point, but osteophyte size does not increase much (except in the Ad-IL-37 group at location 3 in CiOA) and at some locations even decreases. Osteophyte size development is more clearly illustrated in **Suppl. Figure S2**. It shows how differences in growth or shrinkage could explain the increase and decrease of IL-37 effects on osteophyte size at the late time-point

### IL-37 Reduces IL-1β- and OAS-CM-Induced Effects in Human OA Synovial Fibroblasts

Previously, we demonstrated the anti-catabolic effects of IL-37 in human OA chondrocytes.^15,16^ However, in our in vivo experiments we injected IL-37 producing adenoviral constructs into the knee joint, and immune staining showed presence of IL-37 protein in synovial cells, but not in chondrocytes in the articular cartilage. Synovial fibroblasts can also contribute to OA pathology via production of catabolic mediators.^5^ Therefore, we first confirmed in vitro that transduction of human OA fibroblasts with the adenoviral constructs leads to IL-37 mRNA and protein expression, also in the presence of inflammatory mediators **(Suppl. Fig. S3)**. Next, we investigated the functionality of the IL-37 produced by the adenoviral constructs in human OA fibroblast monolayers. Indeed IL-37 could inhibit IL-1β- and OAS-CM-induced pro-inflammatory and proteolytic gene expression. Interleukin-37 significantly reduced both IL-1β-induced *IL1B* and *IL6* expression by 3.5-fold, but did not affect *IL8* expression. Interleukin-37 also significantly reduced OAS-CM-induced *IL6* expression by 4.5-fold **(Suppl. Fig. S4A)**. Next, we investigated the expression of proteolytic cartilage degrading enzymes. Interleukin-37 reduced *MMP1 and MMP3* expression by approximately 2-fold for both IL-1β and OAS-CM respectively **(Suppl. Fig. S4B)**. In contrast, IL-37 did not reduce *MMP13* or *ADAMTS5* gene expression. These data show that adenovirally-delivered IL-37 inhibits *IL1B, IL6, MMP1* and *MMP3* expression in human OA synovial fibroblasts under inflammatory (OA-like) conditions.

## Discussion

In this study, we show that IL-37 reduces OA pathology in two experimental OA models, both in CiOA (females) and DMM (males). Articular cartilage damage was significantly inhibited and also another hallmark of OA, osteophyte development, was clearly diminished. These results establish administration of IL-37 as a potential treatment for OA patients of both sexes.

Previously, we have demonstrated the cartilage protective effects of IL-37 on human OA cartilage chondrocytes and cartilage explants.^15,16^ However, not only chondrocytes are involved in the OA process, but also macrophage-like and fibroblast-like synoviocytes, via the release of catabolic mediators.^6,12^ In the underlying study the intra-articularly injected adenoviral constructs did not infect articular chondrocytes in intact articular cartilage, but immune staining did show IL-37 protein production in synoviocytes. The application of serotype 5 adenoviruses (Ad5) vectors in macrophages is hampered by the absence of the endogenous coxackie adenovirus receptor (CAR), but on the fibroblast, this receptor is present^30,31^ For this reason, we expect that the fibroblasts and not the macrophages of the synovial lining were transfected in our in vivo studies. The suppressive effect of IL-37 on release of inflammatory mediators by macrophages is well described.^17,31^ In contrast, relatively little was known about the role of IL-37 on human OA synovial fibroblasts. In the in vitro part of this study, we found that overexpression of IL-37 in human synovial fibroblasts in an OA-like environment reduces IL1β, IL6, MMP1, and MMP3 expression levels, similar to its described action in macrophages.^17,32,33^ Together, these in vitro results indicate that IL-37 can ameliorate the inflammation-induced catabolic state of multiple joint tissues. Moreover, the in vitro studies show that IL-37 over-expression can still have these effects in spite of the natural presence of IL-37 in human cells, which is increased under inflammatory conditions.^17^

The anti-inflammatory effect of human IL-37 has been demonstrated in various experimental disease models in mice.^21-23^ Interleukin-37 has shown protective effects in arthritis models. For example, in a streptococcal cell wall (SCW)-induced arthritis model, recombinant IL-37 reduced joint inflammation.^23^ In addition, the synovial expression of IL-1β, IL-6 and TNFα was decreased. Also, mice transgenic for human IL-37 showed reduced joint swelling and consequently inflammation in SCW arthritis.^23^ Moreover, in the collagen-induced arthritis (CIA)-model less cartilage damage was observed in presence of IL-37.^34^ In this model, IL-37-adenovirus or control-adenovirus were intra-articularly injected into knee joints of DBA/1J mice 17 and 23 days after first immunization. Mice injected with the IL-37-adenovirus showed significant reduction in cartilage damage and reduced IL-17-driving cytokine production.

However, the effect of IL-37 in experimental OA models has never been studied before, in spite of the presence of inflammation, especially in CiOA.^35^ In our studies in two models for OA, an IL-37-encoding adenovirus was used in order to produce sufficient amounts of IL-37 by the synovial lining of murine knee joints for a prolonged period of time. Intra-articular injection of IL-37-adenovirus resulted in clearly visible synovial IL-37 protein expression in immune stainings. Because mice do not have endogenous IL-37,^36^ we investigated whether this IL-37 expression could induce damage to the joint. We could not detect IL-37-induced pathology after its production in naïve murine knee joints. Also, in other murine models with human IL-37 no pathology or side-effects have been described.^14,21-23^ In our study, the synovial IL-37 protein expression was clearly detectable for at least 1 week after intra-articular injection. Because OA-like pathology in the murine models develops slowly, we decided to repeat the intra-articular injection with the IL-37- or Luc-adenovirus after 2 weeks. The number of injections was limited to two, to prevent an immune response against IL-37 as a foreign protein.^37^

In the CiOA-model, we found that IL-37 reduced the main characteristics of OA pathology: cartilage destruction and osteophyte formation on day 28. However, on day 42 articular cartilage damage was still reduced, but statistical significance was lost. Osteophyte formation was still clearly inhibited at the late time-point. The cause of the reduced effect of IL-37 at the late time-point could be the that it was not present anymore at an effective concentration during the last 2 to 3 weeks before day 42. We were no longer able to clearly detect synovial IL-37 protein expression on day 28. This indicates that the IL-37 protein and its potential protective effects are already lost at this timepoint. More sustained presence of IL-37 might have caused more persistent effects, but this could not be achieved using the adenoviral constructs. Joint dislocations are a common observation in CiOA and they are the result of ligament damage caused by collagenase. As described earlier,^38^ mice with dislocated joints were excluded from analysis, since dislocated joints develop extreme CiOA symptoms that are not anymore comparable to normal CiOA. Excluding the animals with joint dislocations might have induced bias in the results in the CiOA model, because these animals have the strongest effect of the collagenase treatment and theoretically their exclusion leads to lower extent of OA-like pathology in their experimental group. This is not likely to have determined the outcome of our experiments, because at day 28 of CiOA, where we found statistically significant protective effects of IL-37 on cartilage damage, 3 out of 12 mice were excluded in the ad-Luc group and 2 out of 12 in the ad-IL-37 group. This indicates that the protective effect of IL-37 we found is not induced or improved by the exclusions. However, this might play a role in the pathology found at day 42 of CiOA, where 1 out of 12 mice was excluded in the in ad-Luc group and 4 out of 12 in the ad-IL-37 group. Also in the DMM model, where inflammation is less pronounced,^35^ we found statistically significant protection of articular cartilage, but this was found only at the late time-point. At the early time-point (day 28), cartilage damage was low in this slowly developing model, but the protective effect of IL-37 must have been induced in this first period, because after this time-point we could not detect synovial IL-37 protein expression any more. Moreover, IL-37 did reduce osteophyte size at several sites at both time-points. The locations where decreased osteophyte size was found differed between the models. Together, these studies show that IL-37 has the potential to ameliorate OA pathology, also in case of a relatively low inflammatory activity.

Both on day 28 and the later time-point the highest articular cartilage damage was found on the medial femur in the CiOA, while in the DMM cartilage on the medial tibia was most affected. Interestingly, this difference in the location with highest cartilage pathology between the models corresponds with the compartments of the knee joint where largest osteophyte size was found (largest in medial femur in CiOA, versus largest in medial tibia in DMM) and also these were the model-specific sites where clear IL-37 effects on osteophyte size were still found at the late time-point.

Because there is no expression of IL-37 in the models after day 28, one could argue that pathology at the later time-point is less relevant for the study of its protective effect. For assessment of osteophyte size this holds even more, because osteophytes hardly increase in size between early and late time-point and sometimes even get smaller. Although one has to keep in mind that this was measured in two different groups of mice, this would mean that the osteophytes have lost the stimulus to grow. It would be difficult to infer protective effects if this type of pathology does not worsen.

Although both OA models we used are based on mechanical forces caused by ligament damage, we cannot exclude that IL-37-induced inhibition of inflammation has contributed to the protective effects of IL-37 on cartilage damage and osteophyte formation. This is because of the observation we made at day 7 of the CiOA model: reduced joint capsule thickness. In human OA, synovial joint inflammation is associated with disease progression.^39,40^ The reduced synovial thickening we observed early (Day7) in the CiOA model suggests that IL-37 inhibits inflammation and possibly thereby OA pathology. In line with our finding, mice treated with recombinant-IL-37 showed reduced joint swelling, another measure for joint inflammation, in SCW-induced arthritis.^23^ A difference between our study and the SCW-induced arthritis model is that in our study, IL-37 treatment was started after induction of the CiOA-model, whereas in the SCW-induced arthritis model IL-37-treatment was started prior to induction of the model. The observation that IL-37 reduces joint inflammation in both prophylactic and ongoing disease models, suggests that IL-37 might be a potent therapeutic agent to reduce joint pathology in different stages of OA disease.

Our studies do have some limitations. As mentioned, the intra-articular injections of this type of adenoviral constructs could not be repeated after the second injection, because this caused synovitis and proteoglycan depletion in articular cartilage (unpublished observation, now shown in **Suppl. Fig. S5)**. Other methods are needed to obtain more sustained expression of IL-37 in the joint. Another issue is that the CiOA is performed in female mice, because these show less fighting compared to males and also significantly lower incidence of joint displacements (resulting in exclusion of joints from the assessments). The DMM model for OA does not run efficiently in female mice, so males were used for this model. This means that besides differences between models, also gender-related differences could have played a role in the outcome of our experiments. One could also argue that this makes our conclusions even stronger, because we found protective effects of IL-37 in two different models with different inflammatory activity and different sexes.

Anti-inflammatory therapies have previously been applied to OA patients. However, intra-articular injection of for example IL1RA in patients with knee OA did not reduce OA symptoms compared to placebo.^41^ In addition, the effect of TNFα-blockers on OA has also not been proven clinically yet. The human monoclonal anti-TNFα antibody adalimumab is tested in a randomized double-blind placebo-controlled crossover trial in patients with erosive hand OA but did not show any effect on pain or synovitis after 12 weeks compared to placebo.^42^ Furthermore, inhibitors against inducible nitric oxide synthase (iNOS) are developed. Inducible nitric oxide synthase is an enzyme responsible for the production of nitric oxide (NO); a major pro-inflammatory mediator in OA. However, in a 2-year multicenter study of the oral selective iNOS-inhibitor cindunistat (SD-6010) did not slow down the rate of joint space narrowing in patients with symptomatic knee OA nor did it inhibit OA progression.^43^ Most of these studies are based on targeting one specific cytokine. However, it is thought that combining anti-inflammatory approaches is superior to such treatment.^44^ This is exactly the advantage of IL-37; it does not target one specific cytokine but carries out a multifaceted anti-catabolic program.^16,18^ Therefore, applying IL-37 as a broader strategy to inhibit OA pathology increases the chance of successful OA therapy. Interleukin-37 could reduce pathology in OA patients via reducing joint inflammation in both cartilage and synovial fibroblasts or via a direct effect on the cartilage by reducing MMP activity. Taken together, IL-37 is a factor that could reduce both inflammation and joint damage in OA, and this combination ensures that IL-37 is a potential tool for therapeutic approach of a disease for which up till now no cure is available.

## Supplemental Material

sj-docx-1-car-10.1177_19476035251372304 – Supplemental material for Interleukin-37 Ameliorates Articular Cartilage Damage in Two Murine Models of Osteoarthritis Supplemental material, sj-docx-1-car-10.1177_19476035251372304 for Interleukin-37 Ameliorates Articular Cartilage Damage in Two Murine Models of Osteoarthritis by Ellen W. van Geffen, Henk M. van Beuningen, Joyce Aarts, Elly L. Vitters, Wim H.C. Rijnen, Arjen B. Blom, Fons A.J. van de Loo, Esmeralda N. Blaney Davidson, Marije I. Koenders, Arjan P.M. van Caam and Peter M. van der Kraan in CARTILAGE
