# Supplementary material for: Interleukin-37 Ameliorates Articular Cartilage Damage in Two Murine Models of Osteoarthritis
Source: Cartilage. 2025 Sep 19:19476035251372304. Online ahead of print. doi: 10.1177/19476035251372304 (PMC12449308; doi:10.1177/19476035251372304)
Supplement: sj-docx-1-car-10.1177_19476035251372304 – Supplemental material for Interleukin-37 Ameliorates Articular Cartilage Damage in Two Murine Models of Osteoarthritis [file sj-docx-1-car-10.1177_19476035251372304.docx]

***
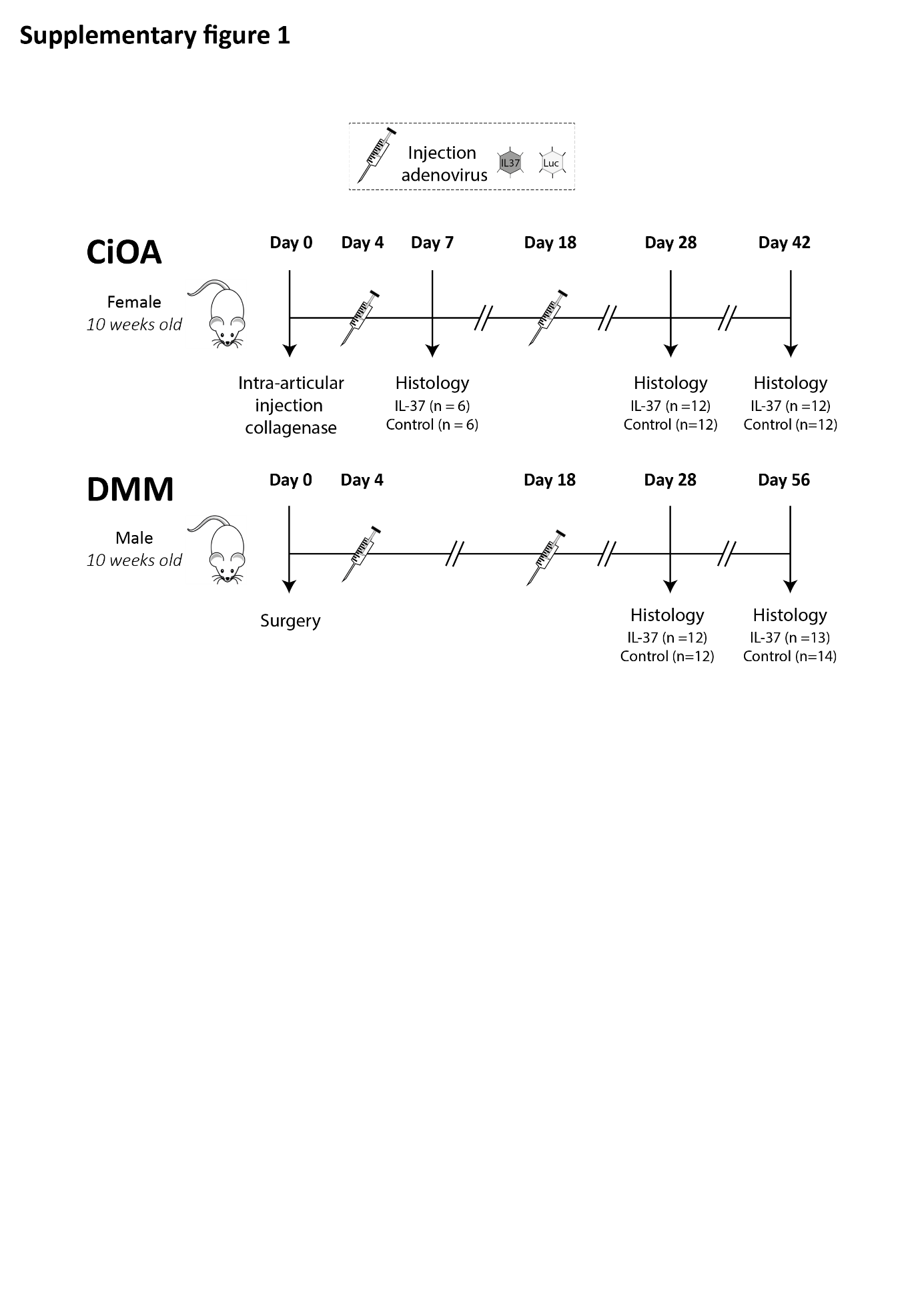
***


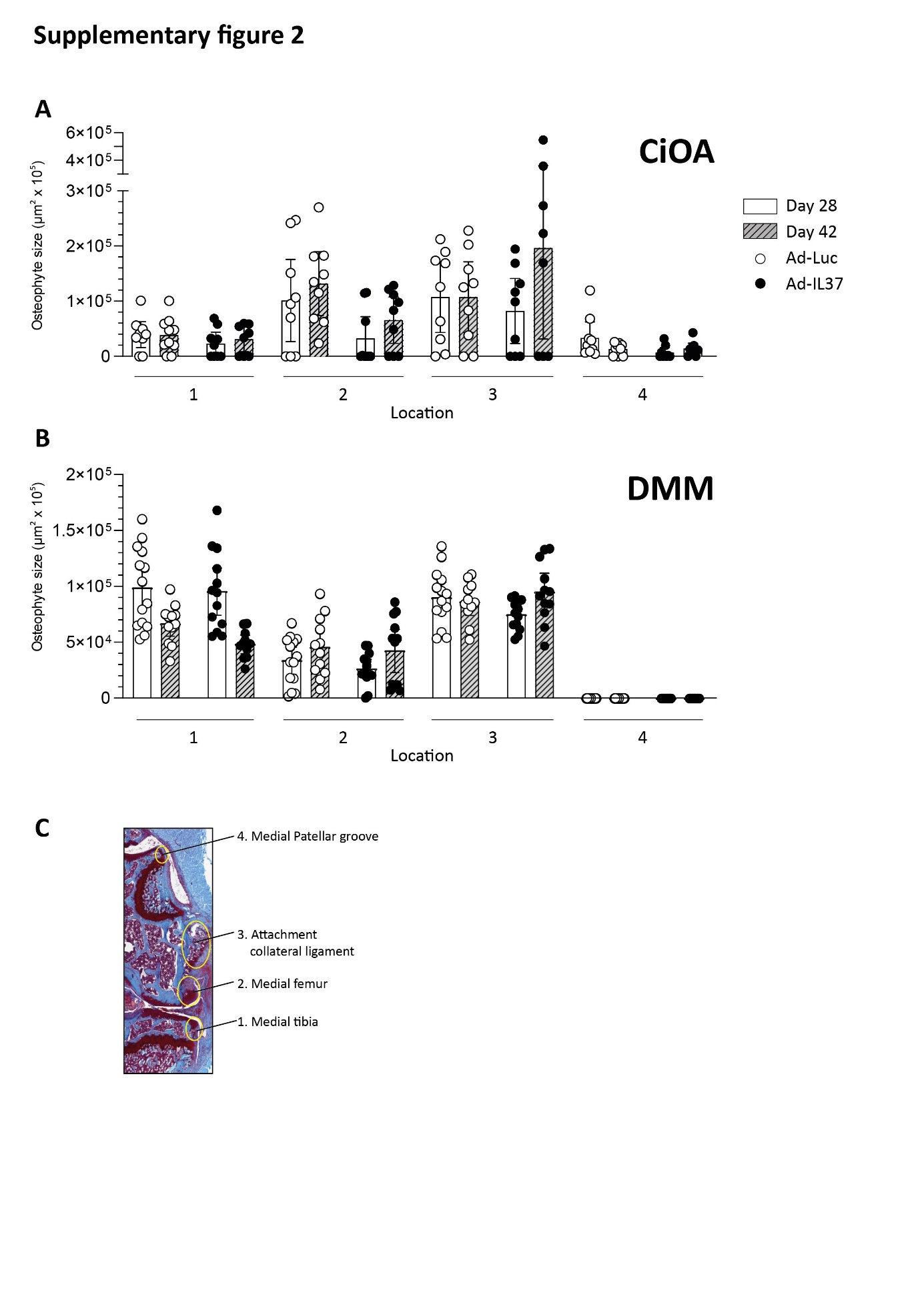


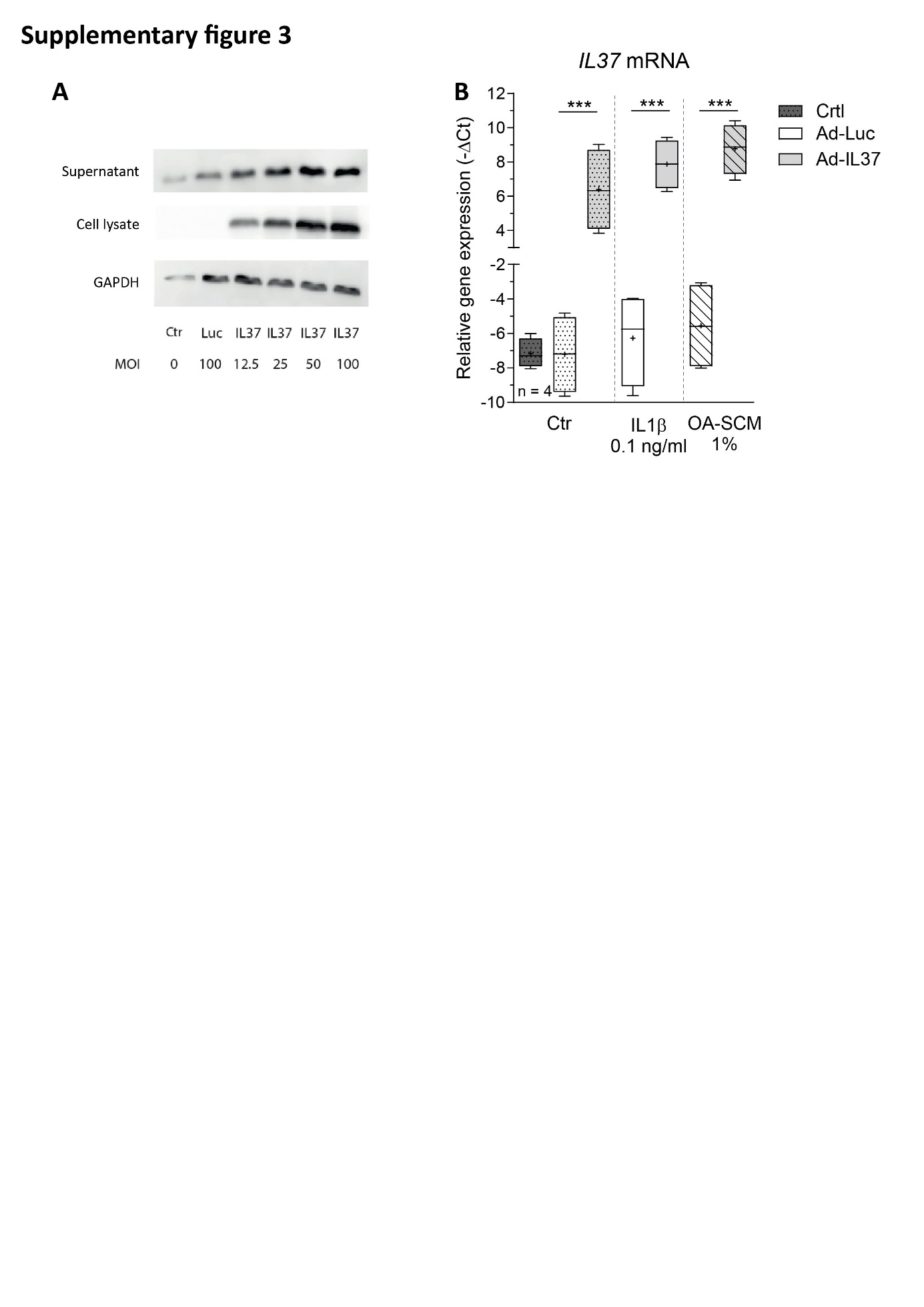


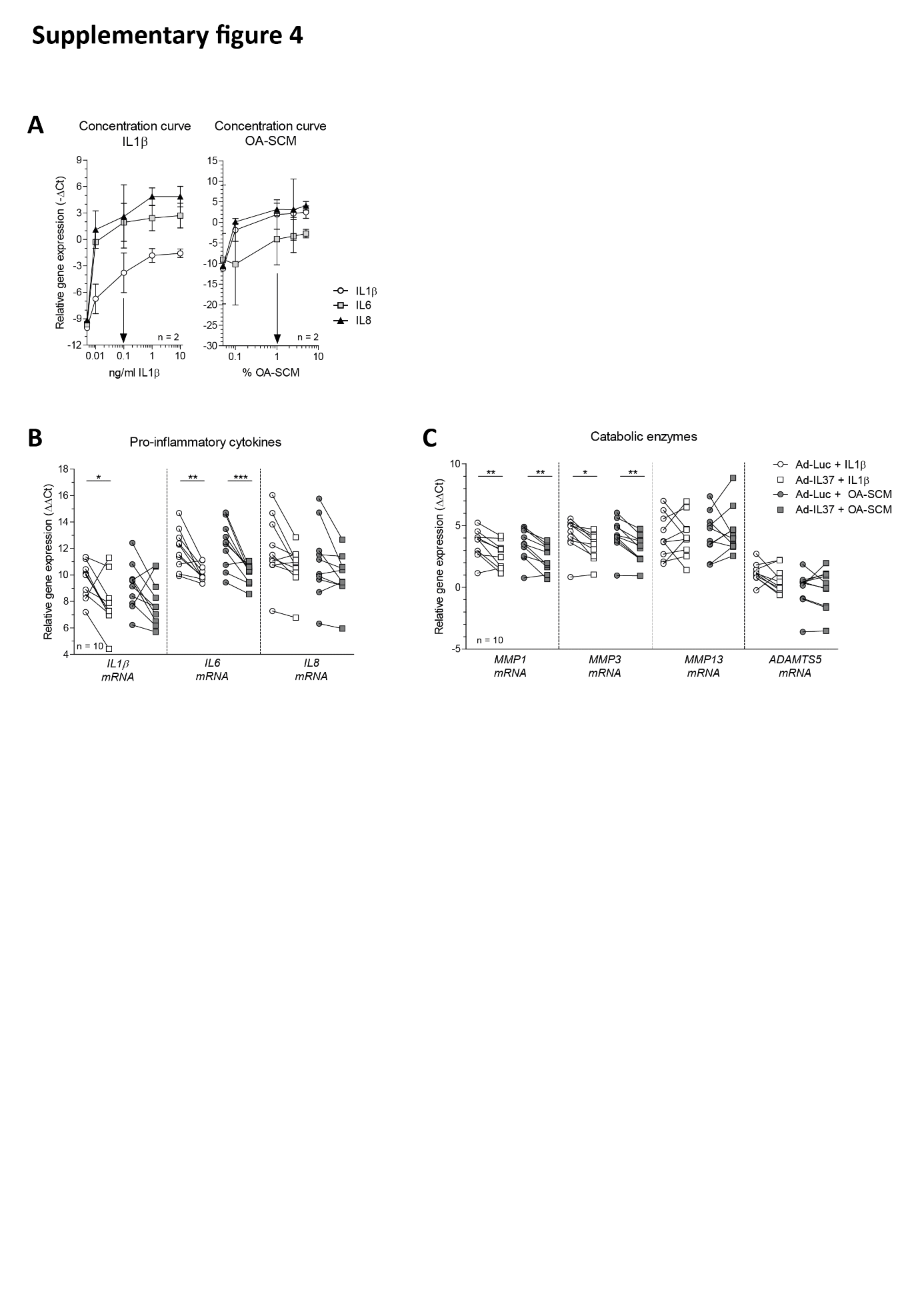


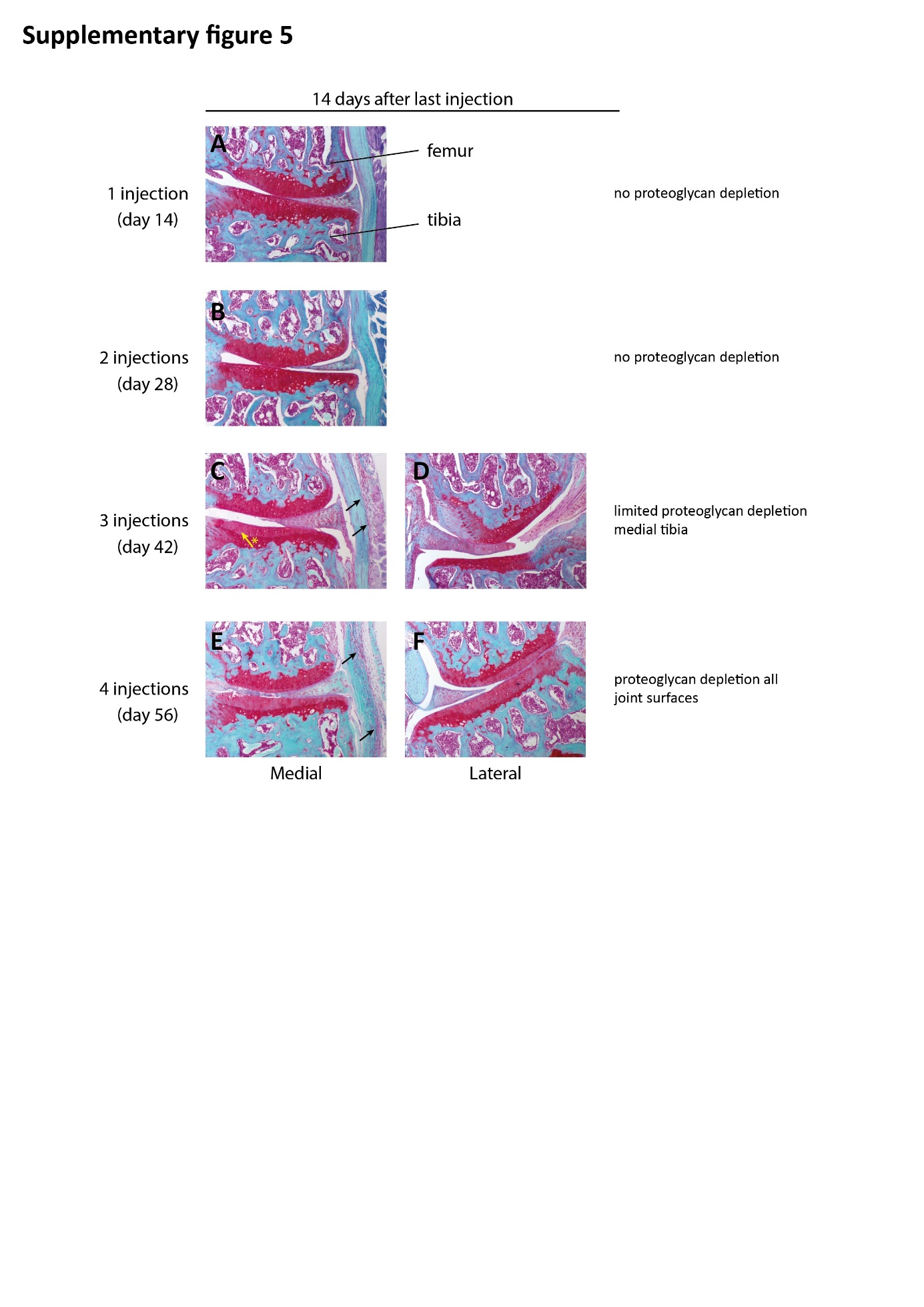


**Supplementary Figure legends**

**Supplementary Figure 1. Overview of the osteoarthritis models.**After arrival in the animal facility and one week of acclimatization OA was induced in two different OA models. **Upper overview**: The collagenase-induced osteoarthritis (CiOA)-model: female C57BL/6NRj mice were intra-articularly injected in the right knee joint with a single injection of 3 units of bacterial collagenase type VII. Four days after collagenase injection, IL-37-adenovirus (Ad-IL-37) or Luciferase-control adenovirus (Ad-Luc) (0.5x10^7^ plaque-forming units (pfu) were intra-articular injected in the right knee joint of mice. Two weeks later, this injection with Ad-IL-37 or Ad-Luc was repeated. The mice were sacrificed on day 7, 28 and 42 after collagenase-injection to be able to perform histological analysis. **Lower overview**: The destabilization of the medial meniscus (DMM) model: This model runs in male C57BL/6NRj mice. Four days after surgery, Ad-IL-37 or Ad-luc was intra-articularly injected, and this was repeated 14 days later. Mice were sacrificed on day 28 and 56 after OA induction to be able to perform histological analysis.

**Supplementary Figure 2. Comparison of osteophyte size at early and late time-points**.

To show the relative change of osteophyte size from early to late time-points this was compared for each experimental group, at 4 different locations, in both models. A) Collagenase-induced OA. B) DMM-induced OA. C) Indication of the 4 different locations in the joint where osteophytes were measured. Data are presented as individual data points with mean ± 95% confidence intervals with each symbol representing one mouse .*= *p*<0.05; ** = p<0.01 as determined by Mann Whitney U-test or unpaired Student’s t-test.

**Supplementary Figure 3. IL-37 expression in human OA synovial fibroblasts after transduction with IL-37-adenovirus.**

To investigate the potency of the IL-37-adenovirus to induce IL-37 protein expression, human OA synovial fibroblasts were transduced with different MOIs (12.5 up to 100) of IL- 37 or Luc-control adenovirus. **A**) IL-37 protein expression in synovial fibroblasts transduced (MOI 12.5-100) with IL-37-adenovirus or Luc-adenovirus as determined by Western blot. In this range, IL-37 expression was MOI-dependently increased with a clear induction in IL-37 protein expression in both cell lysate and supernatant already at a MOI of 25. Plateau level is reached at MOI 50. Because of the strong expression of IL-37 in the cell lysate, this gel needed a very short exposure time, and therefore the endogenous IL-37 in the first 2 lanes is not clearly visible here. **B**) Relative IL-37 gene expression in human OA synovial fibroblasts after IL-37-adenovirus transduction and stimulation with either IL-1β (0.1 ng/ml) or OAS-CM (1%) as determined by qPCR. In combination with either IL1β or OAS-CM stimulation, IL-37 gene expression was even further increased, 2.8-fold and 4.5-fold for respectively IL-1β and OAS-CM, with a significant effect for OAS-CM. Data are presented as box plots with whiskers (min to max), n = 4; *= *p*<0.05; ** = p<0.01; *** = p<0.001 as determined via one-way repeated measures ANOVA with Bonferroni multiple comparison post hoc test.

**Supplementary Figure 4. IL37 decreases pro-inflammatory cytokine and proteolytic enzyme expression in human OA synovial fibroblasts
 A**) Relative gene expression of *IL-1B*, *IL-6* and *IL-8* in human OA synovial fibroblasts after 6 h incubation with IL1β (0-10 ng/ml) or OAS-CM (0-10%) as determined by qPCR (n = 2). **B)** Relative gene expression of pro-inflammatory cytokines *IL-1B*, *IL-6* and *IL-8* in synovial fibroblasts after IL-37-transduction and either IL-1β (0.1 ng/ml) or OAS-CM (1%) stimulation as determined by qPCR. **C)** Relative gene expression of proteolytic enzymes *MMP-1*, *MMP-3*, *MMP-13* and *ADAMTS5* in synovial fibroblasts after IL-37-transduction and either IL1-β (0.1 ng/ml) or OAS-CM (1%) stimulation as determined by qPCR. Data are presented as concentration curves with mean ± 95% confidence intervals (n=2) in A) or aligned dot plots (n = 10 different patients) in B) and C), where lines connect the results in Ad-Luc and Ad-IL-37 transduced cells from the same donor; *= *p*<0.05; ** = p<0.01; *** = p<0.001 as determined via one-way repeated measures ANOVA with Bonferroni multiple comparison post hoc test.

**Supplementary Figure 5. Repeated injections of adenoviral constructs into the murine knee joint induce articular cartilage proteoglycan depletion and synovitis starting from the third injection.** In an earlier (unpublished) experiment we studied the effects of repeated injections of the Ad- luc construct used in the present study, in naïve knee joints. The adenoviral construct was injected with two-weeks intervals and histology was evaluated at two weeks after the last injection, the day that the next injection was given in the remaining animals. A) Medial side of the knee joint at day 14 (one injection). B) Medial side of the knee joint at day 28 (2 injections). C) Medial side of the knee joint at day 42 (3 injections). D) Lateral side of the knee joint at day 42 (3 injections. E) Medial side of the knee joint at day 56 (4 injections). F) Lateral side of the knee joint at day 56 (4 injections). Pictures of Safranin O/fast green stained sections reflect joints of 6 animals per experimental group. In the day 42 group (14 days after the last of three injections) mild proteoglycan depletion (yellow arrow with asterisk) is visible at the medial tibia. In the day 56 (14 days after the last of 4 injections) group articular cartilage proteoglycan loss is visible at the femur and the tibia, both at the medial and the lateral side of the joint. Also synovial inflammation (black arrows) can be observed 14 days after 3 and 4 injections. Based on this histology, in the present study the number of injections was limited to two, to stay on the safe side.
